# Editorial: Technological advances in emergency medical services system, treatment, and prognostication for cardiac arrest

**DOI:** 10.3389/fmed.2023.1145714

**Published:** 2023-03-31

**Authors:** Yasuhiro Kuroda

**Affiliations:** Department of Emergency, Disaster, and Critical care Medicine, Faculty of Medicine, Kagawa University, Miki, Japan

**Keywords:** cardiac arrest, targeted temperature management, REBOA, normoxia, rSO2

This Research Topic on technological advances in cardiac arrest is divided into the following sections: emergency medical system, emergency room treatment to intensive care after ROSC, and prognosis (Figure 1).

**Figure 1:**
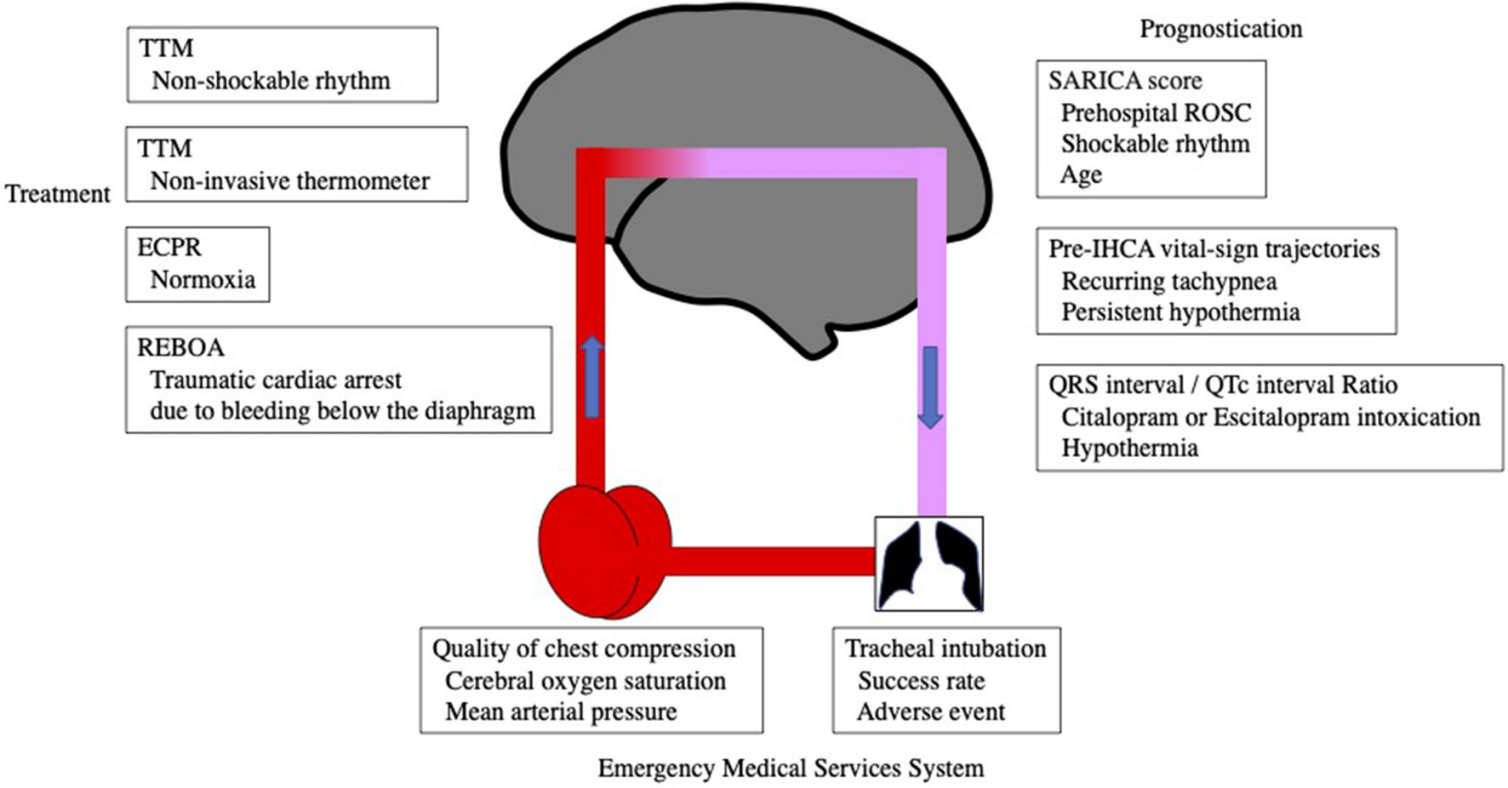
Research topics at the points of cerebral oxygen demand/supply balance.

## Emergency medical system

A first-pass success rate of 85.7%, an adverse event rate of 19.8%, and a muscle relaxant use rate of 5.3% were reported in tertiary care institutions across China for tracheal intubation procedures (Dai et al.). The most common adverse events were hypoxia, hypotension, and aspiration, which were more common in patients with complications such as pulmonary disease and shock. Since only 25% of the patients in this study had cardiac arrest, it is necessary to examine the success rate of tracheal intubation and adverse events in patients with cardiac arrest only, as well as to evaluate outcomes.

The relationship between MAP or SAP from the femoral artery, and cerebral oxygen saturation rSO2 was examined to determine the effect of chest compressions, and it was reported that rSO2 showed a significant association with MAP and SAP (Kishihara et al.) It is possible that rSO2 can non-invasively assess the quality of chest compressions. The challenge is that this report does not provide the rSO2 values necessary for effective chest compressions because the values were evaluated using log transformation and only cases with poor outcome (92% died) were studied. High rSO2 values during CPR have been reported to be associated with cardiac resumption and good neurological outcome (1), and should be discussed with MAP or SAP in the future.

## Intensive care after cardiac resumption from the emergency room

REBOA is used for trauma resuscitation in patients with life-threatening bleeding below the diaphragm (Aoki and Abe). REBOA is contraindicated in TCA patients with chest trauma, i.e., major bleeding from the chest or cardiac tamponade. During cardiopulmonary resuscitation (CPR) in TCA, REBOA increases perfusion of the brain and coronary arteries. REBOA is less invasive than resuscitative thoracotomy with aortic cross-clamping (RT-ACC) and does not require interruption of chest compressions during the procedure. It is important to note that this study notes that REBOA can have serious complications and that future studies are needed to determine the aortic cutoff time of REBOA.

A study of oxygen status in OHCA patients undergoing ECPR found no significant association between hyperoxia in the first 24 h after admission and 30-day survival (Kobayashi et al.). In previous reports of hyperoxia after ECPR being associated with poor outcome, the site of blood gas collection was unknown. In this study, blood gases were drawn from the right radial or right brachial artery, which is significant in that the oxygen status reflects the oxygen status of the brain. Generally, retrograde blood flow from ECMO mixes with retrograde blood flow from the patient's own heart, creating a watershed condition known as Harlequin syndrome (North-South syndrome). Cardiac function was not assessed in this study and it is not known where the watershed is located, and that evaluation is needed in the future.

SpotOn^TM^ creates an isothermal tunnel under the measurement site by applying a probe to the forehead to insulate heat loss from the skin surface, and estimates central body temperature with a zero-heat-flux technology SpotOn^TM^ has been compared to an esophageal temperature probe in patients with cardiac arrest during TTM. Although its accuracy is slightly less than the predetermined 0.5°C, it is a potential method for non-invasively monitoring central body temperature (Fiorini et al.). Previous reports have examined this method over a 9-h monitoring period, but the evaluation of TTM over a 72-h period was obtained in this study, which increases its practicality.

A systematic review showed that the TTM strategy had no significant effect on mortality and neurological outcome in cardiac arrest survivors who presented with an initial non-shockable rhythm (Zhu et al.). However, the greatest weakness of the present study is that it did not distinguish between TTM, i.e., hypothermia, normothermia, fever prevention, and fever left untreated. In addition, although the title of this study indicates that it is an RCT, it is mainly an analysis of observational research articles, and in this sense, there is a lot of bias, which requires caution when interpreting the results.

## Prediction of prognosis

The Survival After ROSC in Cardiac Arrest (SARICA) score (pre-hospital ROSC, age and initial heart rhythm) was shown to predict survival at 30 days with high accuracy in an external validation study using a multinational pan-Asian cohort (Rajendram et al.). The authors comment that the SARICA score is a routinely available and objective variable to evaluate. However, in Japan, serum lactate, pH, cause of arrest, and other factors can be rapidly assessed in the emergency department of a tertiary care hospital, and low-flow or no-flow time can be estimated, even with recall bias, allowing more complex score calculations to assess prognosis (2). On the other hand, a common thread in East Asia is the strong cultural influence regarding the withholding and stopping of life support, which can confound outcomes. This East Asian view differs from that of the West and requires caution when interpreting prognostic assessments.

In a report examining factors that predict the occurrence of IHCA, five factors were reported: SBP, HR, hypothermia, tachypnea, and oxygen saturation. Furthermore, in a multivariate analysis, hypothermia and repetitive tachypnea were independently associated factors as predictors of IHCA (Tsai et al.), suggesting that a better understanding of vital sign changes before IHCA may lead to early detection of deteriorating patients and prevention of IHCA. IHCA is different from OHCA in its cause, and because it is in-hospital, a prompt response can be expected. In this sense, this score is useful.

The QRS interval/QTc interval-ratio has been reported as a predictor of ventricular arrhythmia and cardiac arrest in patients treated with hypothermia or accidental hypothermia. Citalopram and escitalopram, commonly used for depression, often have elevated blood levels, and the QRS/QTc ratio was reported to be predictive of ventricular arrhythmias in these patients (Dietrichs et al.) new score, but future studies are needed to determine how much better it is than the QTc itself.

In this Research Topic, therefore, I will focus on the use of REBOA for traumatic cardiac arrest, control of oxygenation status in ECPR, and factors that predict IHCA (sustained hypothermia, repeated tachypnea).

## Author contributions

The author confirms being the sole contributor of this work and has approved it for publication.
